# Knowledge, experience, and potential risks of dating violence among Japanese university students: a cross-sectional study

**DOI:** 10.1186/1471-2458-11-339

**Published:** 2011-05-19

**Authors:** Mayumi Ohnishi, Rieko Nakao, Satomi Shibayama, Yumi Matsuyama, Kazuyo Oishi, Harumi Miyahara

**Affiliations:** 1 Department of Nursing, Nagasaki University Graduate School of Biomedical Sciences, Nagasaki, Japan; 2 Fukuoka Higashi Medical Centre, Fukuoka, Japan; 3 Nagasaki University Hospital, Nagasaki, Japan

**Keywords:** intimate partner violence, Japanese university student, life skills, health literacy

## Abstract

**Background:**

The Domestic Violence Prevention Act came into effect in Japan in 2001, but covers only marriage partner violence and post-divorce partner violence, and does not recognize intimate partner violence (IPV). The present study was performed to determine the experience of harassment, both toward and from an intimate partner, and recognition of harassment as IPV among Japanese university students.

**Methods:**

A self-administered questionnaire survey regarding the experience of harassment involving an intimate partner was conducted as a cross-sectional study among freshman students in a prefectural capital city in Japan.

**Results:**

A total of 274 students participated in the present study. About half of the subjects (both male and female students) had experience of at least one episode of harassment toward or had been the recipient of harassment from an intimate partner. However, the study participants did not recognize verbal harassment, controlling activities of an intimate partner, and unprotected sexual intercourse as violence. Experience of attending a lecture/seminar about domestic violence and dating violence did not contribute to appropriate help-seeking behavior.

**Conclusions:**

An educational program regarding harassment and violence prevention and appropriate help-seeking behavior should be provided in early adolescence to avoid IPV among youth.

## Background

Intimate partner violence (IPV) is a threat to women's health and is a common concern worldwide [1,2]. In addition, dating violence has been reported in adolescent girls [3]. The lifetime prevalence of physical partner violence and the range of lifetime prevalence of sexual partner violence in Japan were the lowest among the countries included in a multi-country study performed by the WHO in both industrialized and developing countries. Controlling behavior by an intimate partner is one factor associated with physical and IPV, but the percentage of women in Japan who reported such experiences was also the lowest among the countries studied [1,2].

In a study conducted by the Gender Equality Bureau, Cabinet Office, covering the whole of Japan in 2008, 24.9% of female respondents and 13.6% of male respondents reported having experienced physical violence from an intimate partner. The percentage of female respondents who had been recipients of verbal or psychological violence from an intimate partner was 16.8%, while that for males was 8.8%. Among male and female respondents, 8.8% and 16.6% reported having experienced sexual violence from an intimate partner, such as being forced to have sexual intercourse against their will, respectively.

However, legal consideration and intervention programs targeting both married and unmarried young adults are required [4]. The Domestic Violence Prevention Act came into effect in Japan in 2001, but this law covers only marriage partner violence and does not recognize IPV although it does address post-divorce partner violence. In a study among pregnant Japanese women, experience of premarital pregnancy, frequent induced abortion, lack of condom use, and poor education were factors associated with seropositivity for *Chlamydia trachomatis *[5]. Physical violence by an intimate partner was associated with pregnancy, and verbal violence was associated with reduced condom use [6]. There have been no clear surveys regarding dating violence in Japan.

On the other hand, there has been a rapid increase in early sexual debut among high school students and a high prevalence of sexually transmitted infections (STI) and induced abortion among teenagers, as well as a marked decrease in domestic use of condoms in Japan during the 1990s; these changes are thought to be due to the deterioration of human relationships between adolescents/youths and adults, including parents and teachers, and among adolescents/youths themselves [7]. College students in Japan who have had multiple sexual partners show a low likelihood of having used condoms [8]. This also suggests diversification of sexual behavior and an increase in number of casual sex partners among young people over the past two decades in Japan. Thus, the social and family functions of passing wisdom and maturity down from generation to generation appear to be inadequate, and current educational approaches regarding human relationships, including sexuality and sexual education in a school setting, do not appear to be effective in adolescents/youths.

Although the prevalence of self-reported IPV in Japan is not high, there is concern regarding how adolescents/youths establish and maintain relationships with their intimate partners, such as their boy/girlfriend. The present study was performed to determine the experiences of harassment, both toward and receiving from their boy/girlfriend, and recognition of harassment as IPV among freshmen students in a prefectural capital city in Japan. In addition, their consultation and/or help-seeking behaviors were described with regard to IPV. The findings of the present study will contribute to the establishment of educational and support programs to protect human rights and to facilitate healthy relationships with intimate partners among adolescents/youths.

## Methods

### Study participants and study procedure

This was a cross-sectional study performed among freshmen students from two non-medical health faculties (*i.e*., the Faculty of Engineering and Faculty of Environmental Studies) and two medical health faculties (*i.e*., the School of Medicine and School of Health Sciences) from a university in the capital city of one prefecture in Japan in October 2008. Some of the study participants attended a lecture, which was focused on domestic violence (DV) and dating violence as part of optional liberal studies for all faculties' students in the university, and/or had also attended a similar lecture during high school. After obtaining informed consent, the participants completed an anonymous self-administered questionnaire.

### Measures

The self-administered questionnaire solicited information regarding demographic characteristics, experience of harassment toward a boy/girlfriend, experience of receiving harassment from a boy/girlfriend, action and behavior after receiving harassment from a boy/girlfriend, including termination of the relationship, and recognition of harassment as dating violence. Experiences of harassment toward and of receiving harassment from a boy/girlfriend and questions to evaluate recognition of harassment as dating violence were evaluated by 24, 25, and 21 possible episodes of dating violence, respectively. If the study participants recognized the episodes listed in the questionnaire as their own experiences of harassment toward or receiving harassment from an intimate partner and agreed that they constituted harassment, they answered "yes." These episodes consisted of physical, psychological, verbal, sexual, and economic harassment, as well as controlling or limiting the activities of a boy/girlfriend. The questionnaire was prepared based on the booklet "*Do you know about dating violence? *" published by the non-profit organization *DV Prevention Nagasaki*.

### Analysis

Fisher's exact test or the χ^2 ^test was performed to assess the associations with gender and other categorical data, such as age, faculty, experiences of harassment both toward and from a boy/girlfriend, and recognition of harassment as dating violence. Pearson's correlation coefficient and logistic regression analysis were used to assess the association between perpetration and being a victim of harassment. In addition, logistic regression analysis was performed to evaluate factors contributing to consultation-seeking behavior after receiving harassment.

### Ethical clearance

The present study was approved by the Ethical Committees of Nagasaki University Graduate School of Biomedical Sciences. Before completing the questionnaire, all participants gave their informed consent to participation in the study after explanation about its objectives, confidentiality and ethical consideration, and assurance regarding the voluntary nature of participation.

## Results

A total of 274 students participated in the present study. Their demographic characteristics and experiences of having a boy/girlfriend are shown in Table 1. The majority of study participants from non-medical health faculties included students from the Faculty of Engineering and Environmental Sciences, and consisted mainly of male students. The majority of study participants from medical health faculties included students from the Faculty of Nursing, and consisted mainly of female students.

**Table 1:** Demographic information and relationships with the opposite sex (*n *= 274)

	Male (*n *= 126)		Female (*n *= 148)		*P *
		
	*n *	%		%	
Age					
18 years old	44	34.9	61	41.2	
19 years old	62	49.2	62	41.5	
20 years old	20	15.9	25	16.9	
Faculty					
Non-medical health faculty	99	78.6	54	36.5	***
Medical health faculty	27	21.4	94	63.5	
Having relationships with boy/girlfriend					
No	46	36.5	54	36.5	
Yes	80	63.5	94	63.5	

Fisher's exact test. *: *P *< 0.05, **: *P *< 0.01, ***: *P *< 0.001

### Perpetration of abusive behavior

Table 2 shows the types of harassment toward a boy/girlfriend reported by the participants regardless of their recognition of behavior as harassment. Cronbach's alpha for 24 experiences of harassment toward a boy/girlfriend was 0.719. The most frequent type of harassment toward a boy/girlfriend was verbal harassment, followed by checking or controlling the boy/girlfriend's activities. Although the numbers were small, there were reported instances of physical harassment, such as "I have beaten or kicked my boy/girlfriend" (*n *= 2, 1.6% among males and *n *= 1, 0.7% among females), and sexual harassment, such as "I have had sexual relations with my boy/girlfriend against their wishes" (*n *= 3, 2.4% among males).

**Table 2:** Experiences of harassment toward partner by sex (*n *= 274)

	Male (*n *= 126)		Female (*n *= 148)		*P *
		
	*n *	%		%	
I have called my boy/girlfriend "ugly" or "a fool".	30	23.8	41	27.7	
I have asked my boy/girlfriend "What is more important between me and another/others."	6	4.8	7	4.7	
If my boy/girlfriend does not see me, I have said "You don't give me priority."	10	7.9	11	7.4	
I have checked where my boy/girlfriend is and who he/she sees.	18	14.3	15	10.1	
I have become angry because I wanted to know with whom my boy/girlfriend was talking.	26	20.6	18	12.2	
I have meddled with my boy/girlfriend's relationships with friends.	9	7.1	3	2.0	
I have checked my boy/girlfriend's cell phone records without permission from my boy/girlfriend.	1	0.8	5	3.4	
I have demanded my boy/girlfriend delete someone's contact information from his/her cell phone.	5	4.0	1	0.7	
I have deleted someone's contact information from his/her cell phone.	0	0.0	1	0.7	
I have become irritated if my boy/girlfriend disobeys me.	9	7.1	9	6.1	
I have directed to my boy/girlfriend about the place where he/she is going and activities.	7	5.6	11	7.4	
I have blamed my boy/girlfriend if I get angry, because he/she is at fault.	8	6.3	12	8.1	
I have kissed or touched my boy/girlfriend against their wishes.	4	3.2	1	0.7	
I have had sexual relations with my boy/girlfriend against their wishes.	3	2.4	0	0.0	
I have taken money or credit cards from my boy/girlfriend's wallet/purse.	0	0.0	0	0.0	
I have tried to injure myself in front of my boy/girlfriend when unhappy.	2	1.6	1	0.7	
I have injured myself in front of my boy/girlfriend when unhappy.	1	0.8	0	0.0	
I have broken or taken away items precious to my boy/girlfriend.	0	0.0	0	0.0	
I have ignored my boy/girlfriend.	15	11.9	28	18.9	
I have beaten or kicked my boy/girlfriend.	2	1.6	1	0.7	
I have hit something or shouted in front of my boy/girlfriend when angry.	2	1.6	2	1.4	
I have pressed my boy/girlfriend for money.	0	0.0	0	0.0	
I have not returned money borrowed from my boy/girlfriend.	2	1.6	2	1.4	
I have demanded my boy/girlfriend not go to a meeting/party if I am not participating in it.	6	4.8	0	0.0	**

Number and percentages indicate the study participants who responded "yes" to each question.

Fisher's exact test was performed. *: *P *< 0.05, **: *P *< 0.01, ***: *P *< 0.001.

### Prevalence of receiving harassment

Experiences of receiving harassment are shown in Table 3 regardless of the participants' recognition of these experiences as harassment. Cronbach's alpha for 25 experiences of receiving harassment from a boy/girlfriend was 0.827. The most frequent type of harassment received by the participants was verbal harassment, followed by overanxiety regarding where they were and with whom, and free checking of their partner's cell phone records without permission from their partner. Female students were more likely to have checked the cell phone records of their partner without permission and of being afraid of their partner than male students. Although the numbers were small, there were reported instances of physical harassment, such as "I have been beaten or kicked by my boy/girlfriend" (*n *= 1, 0.7% among females), and sexual harassment, such as "I have had sexual relations with my boy/girlfriend against my wishes" (*n *= 2, 1.6% among males and *n *= 3, 2.0% among females).

**Table 3:** Experiences of receiving harassment by sex and recognition of receiving harassment (*n *= 274)

	Male (*n *= 126)		Female (*n *= 148)		*P *
		
	*n *	%	*n *	%	
My boy/girlfriend has called me "ugly" or "a fool."	23	18.3	30	20.3	
I have been hurt, because my boy/girlfriend called me "ugly" or "a fool."	4	3.2	8	5.4	
When I haven't seen my boy/girlfriend, he/she has said "You don't give me priority."	13	10.3	14	9.5	
My boy/girlfriend has persistently contacted me by cell phone.	10	7.9	25	16.9	*
My boy/girlfriend feels anxious where I am and who I see.	17	13.5	28	18.9	
My boy/girlfriend has meddled with my relationships with friends.	5	4.0	10	6.8	
My boy/girlfriend has checked my cell phone records without my permission.	7	5.6	11	7.4	
My boy/girlfriend has demanded I delete someone's contact information from my cell phone.	4	3.2	8	5.4	
My boy/girlfriend has deleted someone's contact information from my cell phone.	0	0.0	3	2.0	
I have felt afraid of my boy/girlfriend.	10	7.9	27	18.2	*
My boy/girlfriend is very kind sometimes, but unkind other times, like a split personality.	4	3.2	12	8.1	
My boy/girlfriend has blamed me if he/she gets angry because I am at fault.	3	2.4	6	4.1	
I have had sexual relations with my boy/girlfriend against my wishes.	2	1.6	3	2.0	
My boy/girlfriend has kissed or touched me against my wishes.	1	0.8	7	4.7	
My boy/girlfriend has taken money or credit cards from my wallet/purse.	1	0.8	0	0.0	
My boy/girlfriend has said "What is more important between me and another/others."	4	3.2	10	6.8	
I have been placed under confinement by my boy/girlfriend.	0	0.0	0	0.0	
My boy/girlfriend has tried to injure himself/herself in front of me when unhappy.	1	0.8	1	0.7	
My boy/girlfriend has injured himself/herself in front of me when unhappy.	1	0.8	1	0.7	
My boy/girlfriend has broken or taken away items precious to me.	1	0.8	1	0.7	
I have been ignored by my boy/girlfriend.	10	7.9	11	7.4	
I have been beaten or kicked by my boy/girlfriend.	0	0.0	1	0.7	
My boy/girlfriend has hit something or shouted in front of when angry.	2	1.6	2	1.4	
I have been pressed for money by my boy/girlfriend.	0	0.0	1	0.7	
My boy/girlfriend has borrowed money from me and not returned it.	1	0.8	1	0.8	

Number and percentages indicate the study participants who responded "yes" to each question.

Fisher's exact test. *: *P *< 0.05, **: *P *< 0.01, ***: *P *< 0.001.

Pearson's correlation coefficient between having at least one episode of perpetration of any type of harassment and being the victim of any type of harassment was 0.708 (*P *< 0.001). Regardless of sex, age, faculty, and experiences of attending a lecture/seminar about DV and/or dating violence, having at least one episode of perpetration of any type of harassment was associated with having at least one episode of being a victim of any type of harassment (adjusted odds ratio: 37.864, 95% confidence interval: 18.593, 77.112, *P *< 0.001).

### Help-seeking behavior after receiving harassment

A total of 131 participants reported having at least one episode of receiving harassment, including physical, verbal, and sexual harassment, from a boy/girlfriend, and 121 participants responded to the questions regarding seeking consultation or help after receiving harassment. Only 42 (34.7%) participants (9 males and 33 females) sought any type of consultation or help after receiving harassment, and there was a significant difference between male and female students, with female participants being more likely to seek consultation or help after receiving harassment (Fisher's exact test, *P *< 0.01). Most reported consulting with their friends (8 male students and 32 female students). Six female students sought help from their mother, 5 sought help from their sister, and one sought help from health personnel. Only two male students sought help from their brother, and none sought help from parents after receiving harassment. None of the participants reported seeking help from a teacher, school nurse, or women's welfare consultation center that provides services of consultation and help for women with harassment and violence. Only one female victim reported that she had consulted with a medical health professional after harassment. Seventy-nine (65.3%) participants did not seek any type of consultation or help after receiving harassment. Seventy-eight participants (39 males and 39 females) responded to the question(s) regarding the reason(s) why they did not seek any consultation or help as follows: they felt that the episode was not serious enough to warrant consultation (35 males and 33 females); they felt that they also had some responsibility in the episode (10 males and 6 females); and they felt that the episode was an expression of love from their boy/girlfriend (5 males and 8 females).

### Preference of help-seeking after receiving harassment

A total of 268 participants (97.8%; 121 males and 147 females) reported having heard about "dating violence," defined as harassment or violence in a non-married couple: 234 (85.4%) from "the media," 152 (55.5%) from "school/university lecture," 29 (10.6%) from "friends," 16 (5.8%) from "seminar outside of school/university," and 13 (4.7%) from "family." A total of 114 participants (41.6%; 38 males and 76 females) reported having heard about "dating violence": 78 (28.5%) from "the media," 58 (21.2%) from "school/university lecture," 9 (3.3%) from "friends," 6 (2.2%) from "seminar outside of school/university," and 2 (0.7%) from "family."

Among 268 participants who had heard about dating violence, 135 (50.4%; 47 males and 88 females) responded to the question(s) concerning their preference regarding person/organization from which to seek consultation or help in the event of a future episode of dating violence, while 133 (49.6%) did not provide responses to questions regarding preference for seeking consultation or help. Among 135 participants who expressed a preference regarding person/organization from which to seek consultation or help, the most frequent answer was "friend" among both male and female students (39 and 82, respectively), followed by "mother" and "counseling center," while a few mentioned "father," "sister," "brother," "police," "health care personnel," "teacher," "school nurse," "school counselor," and "women's welfare consultation center." On logistic regression analysis, regardless of age and faculty, female gender (*P *< 0.001) showed a significant positive independent association, while experiences of attending a lecture/seminar about DV and dating violence (*P *< 0.01) showed a significant negative independent association with seeking consultation or help after experiencing harassment.

### Recognition of abusive behavior

Cronbach's alpha for 21 possible episodes to evaluate recognition of harassment was 0.549. As shown in Table 4 more than half of the participants did not recognize verbal harassment or checking and controlling their boy/girlfriend's activities as harassment. Compared to female students, male students were less likely to recognize sexual intercourse without use of contraception to guard against unwanted pregnancy and condoms to protect against STIs (Fisher's exact test, *P *< 0.001) or taking money from their girlfriend's wallet without permission (Fisher's exact test, *P *< 0.001) as harassment. More than 20% of male students responded "no" to the question "I think harassment is a bad behavior." On the other hand, more than 20% of females felt that if their relationship with their boyfriend was on intimate terms, she should comply with a request for sexual relations even if she did not want to engage in sexual activity. More than 80% of males and more than 50% of females responded that they would not terminate their relationship despite violence, and the difference between males and females was statistically significant (Fisher's exact test, *P *< 0.001).

**Table 4:** Recognition of attitudes and behaviors as violence, including dating violence (*n *= 274)

	Sex					Faculty					Experience of lecture about DV or dating DV				
	
	Male (*n *= 126)		Female (*n *= 148)		*P *	Non-medical health (*n *= 153)		Medical health (*n *= 121)		*P *	No (*n *= 110)		Yes (*n *= 164)		*P *
						
	*n *	%	*n *	%		*n *	%	*n *	%		*n *	%	*n *	%	
I think that date DV is a problem only for older people.	123	97.6	147	99.3		150	98.0	120	99.2		108	98.2	162	98.8	
I think that beating or kicking boy/girlfriend is violence. (#)	104	82.5	130	87.8		121	79.1	113	93.4	**	91	82.7	143	87.2	
I think calling my boy/girlfriend "ugly" or "a fool" is violence. (#)	63	50.0	70	47.3		67	43.8	66	54.5		42	38.2	91	55.5	**
If I control my boy/girlfriend's activities and his/her human relationships, it is violence. (#)	39	31.0	66	44.6	*	48	31.4	57	47.1	**	31	28.2	74	45.1	**
If my boy/girlfriend does not collaborate in prevention of unwanted pregnancy, it is violence. (#)	70	55.6	121	81.8	***	90	58.8	101	83.5	***	60	54.5	131	79.9	***
I think having a sexual relationship without protection against sexually transmitted infections is violence. (#)	65	51.6	115	77.7	***	79	51.6	101	83.5	***	59	53.6	121	73.8	**
I think free taking of money from my boy/girlfriend's wallet or pressing for money is violence. (#)	72	57.1	106	71.6	*	82	53.6	96	79.3	***	59	53.6	119	72.6	**
I think harassment is a bad behavior. (#)	98	77.8	131	88.5	*	116	75.8	113	93.4	***	85	77.3	144	87.8	*
I think a recipient of violence also bears some responsibility. (#)	27	21.4	29	19.6		28	18.3	28	23.1		18	16.4	38	23.2	
I think violence occurs due to drinking alcohol and/or becoming irritated.	95	75.4	104	70.3		116	75.8	83	68.6		84	76.4	115	70.1	
I think people subject their boy/girlfriend to harassment/violence because they do not love him/her.	113	89.7	141	95.3		140	91.5	114	94.2		102	92.7	152	92.7	
I think it is possible to avoid unwanted sexual relations if one really does not want to engage in sex.	90	71.4	114	77.0		110	71.9	94	77.7		82	74.5	122	74.4	
Although there may be violence between a couple, it is not recognized as violence if they are a committed couple.	115	91.3	140	94.6		140	91.5	115	95.0		110	91.8	154	96.9	
I will terminate a relationship with my boy/girlfriend if he/she is violent. (#)	19	15.1	62	41.9	***	31	20.3	50	41.3	***	31	28.2	50	30.5	
If a couple with violence does not terminate their relationship, the violence is not serious.	115	91.3	141	95.3		140	91.5	116	95.9		101	91.8	155	94.5	
I think it is possible to avoid violence depending on my behavior.	108	85.7	131	88.5		135	88.2	104	86.0		97	88.2	142	86.6	
I think my boy/girlfriend should be forgiven if he/she apologizes after being violent.	110	87.3	137	92.6		134	87.6	113	93.4		110	90.9	147	89.6	
My boy/girlfriend does not like when I talk or go out with others, because he/she loves me.	99	78.6	106	71.6		127	83.0	78	64.5	***	88	80.0	117	71.3	
If a relationship is on intimate terms, I should comply with requests for sexual relations.	113	89.7	144	97.3	*	139	90.8	118	97.5	*	104	94.5	153	93.3	
The risk of pregnancy is low if I have a sexual relationship without protection, and it is therefore not necessary to use contraception.	122	96.8	145	98.0		148	96.7	119	98.3		106	96.4	161	98.2	*
If my boy/girlfriend loves me, I should comply with their requests for sexual intercourse even if I do not want to engage in sex.	121	96.0	143	96.6		146	95.4	118	97.5		107	97.3	157	95.7	

Numbers and percentages indicate the study participants who responded "no" to each question without (#) and "yes" to each question with (#) regarding recognition of attitude and behavior as harassment, abuse, or violence.

Fisher's exact test was performed. *: *P *< 0.05, **: *P *< 0.01, ***: *P *< 0.001.

## Discussion

The most common form of harassment--both toward and from a boy/girlfriend--was verbal harassment, and about half of the participants of both genders did not recognize this as harassment. This was followed by checking and/or controlling the activities and behavior of an intimate partner, and free checking of the intimate partner's cell phone records without permission. The number of reports of having sexual relations against their will was not high, but there were instances of such cases. In both genders, about 80% of the participants felt that the recipient of harassment also shared responsibility, and thus the recipients also had a negative aspect, and about half or more participants did not recognize verbal harassment and checking and/or controlling the activities and behavior of an intimate partner as violence. Almost half of the male participants did not recognize lack of collaboration in using contraception to protect against unwanted pregnancy and STIs or freely taking money from their girlfriend's purse as violence. The participants with experience of harassment toward an intimate partner showed an association with experience of receiving harassment, and a higher rate of receiving harassment influenced seeking consultation or help.

The results of the present study showed that most of the freshman students had appropriate knowledge allowing them to recognize harassment as IPV. However, male students were especially lacking in knowledge regarding safe sex with protection against unwanted pregnancy and STIs. Lack of consideration of the outcomes of unprotected sexual relationships represents a type of violence. A study in the USA indicated that men reporting traditional ideologies were more likely to report unprotected vaginal intercourse and IPV toward their female partner [9], and men with experience of IPV toward their partner were more likely to show unhealthy and unprotected sexual behavior, such as forcing sexual intercourse and having multiple sexual partners [10]. The Safe Date Program implemented among adolescents in the USA addressed sexual health in dating relationships. However, evaluations performed to date have not demonstrated long-term effectiveness to reduce risks to adolescent sexual health [11]. To avoid unwanted outcomes from unprotected sexual relationships, comprehensive educational approaches in the early adolescent period are needed, for both male and female students.

In the present study, higher rates of harassment toward a boy/girlfriend were associated with higher rates of receiving harassment from a boy/girlfriend. A previous study regarding IPV among young adults indicated that there is an interaction between perpetration and being a victim of harassment [12,13]. Reciprocal dating violence is common, which often leads to injury among adolescents [14]. There have been several discussions regarding high-risk behaviors, power balance in couples, and motivation and outcome of reciprocal and non-reciprocal dating violence among adolescents and youth [15-17]. An appropriate understanding of IPV requires not only a focus on gender-related differences and physical/sexual violence, but background environmental and social and cultural norms must also be taken into consideration [18]. Although female students were more likely to seek consultation and/or help after receiving harassment, the present study did not compare reporting from male and female sides of the same couples or the sociocultural environments of the study participants. Therefore, it was not possible to appropriately discuss the motivations underlying harassment or the depth and/or severity of harassment, especially considering gender differences. The power balance in couples, depth of outcomes of harassment, and background of IPV should be examined in future studies.

In addition, IPV was associated with limitation of partner's activities [19,20], and higher levels of risk behavior, such as antisocial or violent behavior, among adolescents [21,22]. The present study showed that the most frequent types of harassment were verbal harassment, checking and/or controlling intimate partner activities and behaviors, forcing unprotected sexual intercourse, and taking money from an intimate partner's wallet or purse without permission. Most of the participants did not recognize these types of harassment as violence. These results suggest that there is an association between ignorance and more severe violence among Japanese freshmen students. Violent relationships with intimate partners, including verbal violence and ignoring their partner, can result in the accumulation of severe violence and unfavorable outcomes, such as unwanted pregnancies and STIs. It is important to provide an opportunity for educational intervention with regard to intimate partner harassment and violence to protect and promote Japanese freshman students' health and rights in human relationships during the teenage period regardless of gender and faculty.

The present study demonstrated that there is an adverse association between the experiences of attending a lecture/seminar about DV and dating violence and seeking of consultation and/or help after receiving harassment. That is, the experience of attending a lecture/seminar about DV and dating violence did not promote appropriate help-seeking behavior. Only about 60% of women in rural Vietnam with injuries due to IPV sought health care [23]. Educational programs for Japanese freshmen should include not only knowledge about harassment and violence, but also information about where appropriate consultation and/or help, including help from medical health professionals, can be found for those exposed to harassment and violence.

There present study had several limitations. First, this was a cross-sectional study, and Cronbach's alpha for IPV knowledge was not strong, although that for experiences of harassment toward and of receiving harassment from a boy/girlfriend was relatively high. Therefore, the results cannot be interpreted to determine causality. Second, data were collected by self-reporting about experiences of harassment. Therefore, the data were subject to recall bias and under-or overstatement, and so may deviate from reality. Future studies at the level of individual couples will yield a greater understanding of adolescent and youth dating violence. Third, the participants were recruited from a university located in the capital city of one prefecture in Japan. The social norms and environment, including faculty and past exposure to educational programs regarding violence, may influence knowledge and information available, as well as judgment of harassment and violence. Therefore, the results cannot be taken as generally representative of Japanese youth. Although there were limitations, the present study afforded provided a better understanding regarding potential risks of harassment and violence among Japanese youth, and thus suggested a basis for possible interventions.

## Conclusion

The number of educational programs regarding harassment and violence for Japanese youth has increased over the past decade. However, the results of the present study indicated that improvements are still necessary in awareness and understanding regarding harassment and violence among Japanese youth. Educational programs dealing with harassment and violence prevention should be provided in early adolescence, and should include information regarding different types of harassment and violence, including physical, sexual, psychological, and economic violence, as well as protective measures, including appropriate behavior regarding seeking help in the event of becoming a victim. Not only specific educational programs regarding harassment and violence, but also general programs for improvement of life skills and health literacy among Japanese youth should be provided in several settings, such as school/university and community. In addition, it is necessary to integrate formal and academic education at school and university and life skills programs to protect and promote the health of youth.

## Competing interests

The authors declare that they have no competing interests.

## Authors' contributions

MO supervised planning and procedure of the study, was responsible for data analysis and wrote the manuscript. RN, TS, and YM prepared a draft of the study proposal and were responsible for data collection. KO and HM contributed to conceptualization of the study and made revisions to the manuscript before submission. All authors read and approved the final manuscript.

## Pre-publication history

The pre-publication history for this paper can be accessed here:

http://www.biomedcentral.com/1471-2458/11/339/prepub
